# Loss of forebrain MTCH2 decreases mitochondria motility and calcium handling and impairs hippocampal-dependent cognitive functions

**DOI:** 10.1038/srep44401

**Published:** 2017-03-09

**Authors:** Antonella Ruggiero, Etay Aloni, Eduard Korkotian, Yehudit Zaltsman, Efrat Oni-Biton, Yael Kuperman, Michael Tsoory, Liat Shachnai, Smadar Levin-Zaidman, Ori Brenner, Menahem Segal, Atan Gross

**Affiliations:** 1 Department of Biological Regulation, Weizmann Institute of Science, Rehovot 7610001, Israel; 2 Department of Neurobiology, Weizmann Institute of Science, Rehovot 7610001, Israel; 3 Department of Veterinary Resources, Weizmann Institute of Science, Rehovot 7610001, Israel; 4 Department of Chemical research Support, Weizmann Institute of Science, Rehovot 7610001, Israel

## Abstract

Mitochondrial Carrier Homolog 2 (MTCH2) is a novel regulator of mitochondria metabolism, which was recently associated with Alzheimer’s disease. Here we demonstrate that deletion of forebrain MTCH2 increases mitochondria and whole-body energy metabolism, increases locomotor activity, but impairs motor coordination and balance. Importantly, mice deficient in forebrain MTCH2 display a deficit in hippocampus-dependent cognitive functions, including spatial memory, long term potentiation (LTP) and rates of spontaneous excitatory synaptic currents. Moreover, MTCH2-deficient hippocampal neurons display a deficit in mitochondria motility and calcium handling. Thus, MTCH2 is a critical player in neuronal cell biology, controlling mitochondria metabolism, motility and calcium buffering to regulate hippocampal-dependent cognitive functions.

Brain tissue has exceedingly high-energy demands compared to all other organs^1^. Mitochondria are key organelles that allow efficient energy production to support neuronal functions, including the mobilization of synaptic vesicles for exocytosis and recycling, the assembly of the actin cytoskeleton for presynaptic development and the maintenance of neuronal membrane potential^2^. In addition to carrying out aerobic respiration to generate ATP, mitochondria function actively in the buffering of intra-cellular Ca^2+^ concentration and in the mechanisms of apoptosis^3^. Thus, mitochondria need to move freely and distribute properly throughout the axodendritic domains to enable neuronal activity^4^,^5^. When mitochondrial functionality is compromised, changes in cellular energetics are observed and whole-body energy balance is disturbed.

Mitochondrial carrier homolog 2 (MTCH2) is a novel mitochondrial outer membrane protein that acts as a receptor-like protein for the BH3 interacting-domain death agonist (BID) and is essential for induction of apoptosis in the liver^6^. In recent years, many genome-wide association studies have correlated single nucleotide polymorphism (SNP) in the MTCH2 gene with increased body mass index, obesity and diabetes^7^,^8^,^9^,^10^,^11^,^12^. Knocking out MTCH2 in mice results in lethality at embryonic day 7.5, suggesting that MTCH2 plays a critical role in embryonic development^6^.

Recently, we have shown that mice deficient in skeletal muscle MTCH2 are protected from diet-induced obesity, and that they demonstrate an increase in whole-body energy metabolism^13^. In that study and in an earlier one performed in the hematopoietic system^14^ we also showed that loss of MTCH2 increases many parameters related to mitochondria function including oxidative phosphorylation (OXPHOS), mitochondrial size, and the levels of ATP, NADH and reactive oxygen species (ROS). Taken together, these findings indicate that MTCH2 is a pivotal regulator of mitochondria and whole-body energy metabolism.

Abnormal mitochondrial morphology and function are associated with neurodegenerative diseases^3^,^15^,^16^,^17^ and defects in learning and memory^18^,^19^,^20^,^21^,^22^. Interestingly, MTCH2 locus shows gene-based genome-wide significant association with Alzheimer’s disease (AD)^23^,^24^,^25^, and MTCH2 expression levels decrease in AD cases in parallel with the severity of the disease^24^. Mitochondrial dysfunction is considered to be a trigger for the pathogenesis of AD^16^,^26^,^27^,^28^. The hippocampal formation is recognized for its pivotal role in memory formation, and is one of the earliest regions in the brain to be affected in AD. Interestingly, the hippocampal formation and the cerebellum are the regions in the brain with the highest MTCH2 expression^29^,^30^.

To elucidate the role of MTCH2 in cognitive functions, through its control of mitochondria, we chose to study the effect of its selective deletion in the forebrain.

## Results

### *MTCH2* ^*F/F*^ *CamKIIα-Cre* ^+^ mice display increased mitochondria and whole-body energy metabolism, increased locomotor activity, but impaired motor coordination and balance

**Figure 1:**
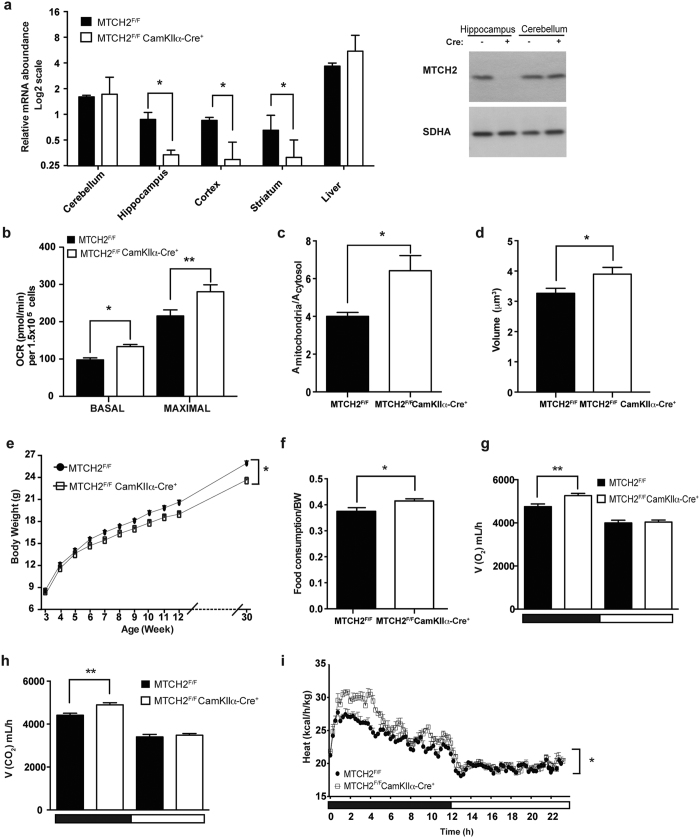
*MTCH2*^*F/F*^*CamKIIα-Cre*^+^ mice display increased mitochondria and whole-body energy metabolism. (**a**) Specific MTCH2 knockout in the forebrain. Left panel: relative gene expression evaluated by real-time PCR (normalized to HPRT expression) using lysates from cerebellum, hippocampus, cortex, striatum, and liver of *MTCH2*^*F/F*^and *MTCH2*^*F/F*^*CamKIIα-Cre*^+^ mice. Results are presented as mean relative expression ± SEM (*p ≤ 0.05, n = 3 mice). Right panel: Western blot analysis using anti-MTCH2 Abs of lysates prepared from hippocampus and cerebellum of *MTCH2*^*F/F*^ and *MTCH2*^*F/F*^ *CamKIIα-Cre*^+^ mice. Succinate Dehydrogenase A (SDHA) served as loading control. (**b**) Loss of forebrain MTCH2 increases oxygen consumption. Normalized average of oxygen consumption rate (OCR) measured in real-time indicating basal and maximal respiration rates in XF media containing 10 mM Pyruvate (as indicated by OCR before and after injection of FCCP). The data represent mean ± SEM (*p ≤ 0.05; **p ≤ 0.01) of 4 independent experiments. (**c**) Mitochondrial area is increased in the soma of *MTCH2*^*F/F*^*CamKIIα-Cre*^+^ neurones. The data represent mean ± SEM (*p ≤ 0.05; n = 3 mice/each genotype; n = 40 neurons/each mouse). (**d**) Mitochondria volume is increased in axons and dendrites lacking MTCH2. Morphometric analysis of 3D reconstructed mitochondria of neurons transfected with MitoDsRed. Total volumes of axonal mitochondria of *MTCH2*^*F/F*^ and *MTCH2*^*F/F*^ *CamKIIα-Cre*^+^ primary hippocampal neurons are shown. The data represent mean ± SEM (*p ≤ 0.05, 3 independent experiments, 30 fields). (**e**) *MTCH2*^*F/F*^ *CamKIIα-Cre*^+^ mice gain less weight on chow diet. The weight gain of the *MTCH2*^*F/F*^ and *MTCH2*^*F/F*^ *CamKIIα-Cre*^+^ littermate mice fed on chow diet for 30 weeks are presented. The data represent mean ± SEM (*p ≤ 0.05, n = 12–35 mice; Two-way ANOVA). (**f**) *MTCH2*^*F/F*^ *CamKIIα-Cre*^+^ mice eat more on chow diet. Sum of 24 h food intake measured while housed in the metabolic cages. The data represent mean ± SEM (*p ≤ 0.05, n = 8 mice). (**g**) *MTCH2*^*F/F*^ *CamKIIα-Cre*^+^ mice show increased oxygen consumption. The data represent mean ± SEM (**p ≤ 0.01, n = 8 mice). The light and dark cycles are denoted by horizontal white and black bars, respectively. (**h**) *MTCH2*^*F/F*^ *CamKIIα-Cre*^+^ mice show increased CO_2_ production. The data represent mean ± SEM (**p ≤ 0.01, n = 8 mice). The light and dark cycles are denoted by horizontal white and black bars, respectively. (**i**) *MTCH2*^*F/F*^ *CamKIIα-Cre*^+^ mice show increased heat production. The data represent mean ± SEM (*p ≤ 0.05, n = 8 mice). The light and dark cycles are denoted by horizontal white and black bars, respectively.

To determine the relevance of the genome-wide association studies described earlier in an *in vivo* setting, we generated a genetic mouse model where MTCH2 deletion is driven by the α subunit of the calcium/calmodulin-dependent protein kinase IIα (CamKIIα) Cre allele^31^ (*MTCH2*^*F/F*^*CamKIIα-Cre*^+^) resulting in specific MTCH2 knockout in excitatory neurons of the hippocampus, cortex and striatum (Fig. 1a, left and right panels). The *MTCH2*^*F/F*^ *CamKIIα-Cre*^+^ mice are viable with no obvious brain histological abnormalities; however, the males are infertile because of severe testis degeneration and reduction of mature spermatozoa [Supplementary Fig. S1a; the Cre-recombinase was found to be expressed also in the testis (data not shown) resulting in decreased MTCH2 levels; Supplementary Fig. S1b].

Two of the hallmarks of MTCH2 deficiency in the bone marrow and skeletal muscle are an increase in mitochondrial OXPHOS and an increase mitochondria size/volume^13^,^14^. To assess whether a similar impairment in OXPHOS occurs as a consequence of forebrain MTCH2 deletion, we measured cellular respiration in primary hippocampal neuronal cultures, and found that loss of MTCH2 led to an increase in both basal and maximal oxygen consumption (Fig. 1b, and Supplementary Fig. S1c). To assess the effect of forebrain MTCH2 deletion on mitochondria size, we conducted electron microscopic analysis in the CA1 neurons of the hippocampus. These analyses indicated that mitochondria in the soma of *MTCH2*^*F/F*^ *CamKIIα-Cre*^+^ neurones were significantly enlarged (Fig. 1c and Supplementary Fig. S1d,e). In addition, three-dimensional (3D) volumetric analysis of dendritic mitochondria by computerized segmentation showed that *MTCH2*^*F/F*^*CamKIIα-Cre*^+^ primary hippocampal neurons have increase mitochondrial volume as compared with the control *MTCH2*^*F/F*^neurons (Fig. 1d). Interestingly, there was no difference in the number of mitochondria per cell in the soma of the CA1 neurons (Supplementary Fig. S1f), which is consistent with the lack of difference in mitochondrial DNA (mtDNA; Supplementary Fig. S1g).

Next, we examined whole-body energy metabolism and found that the *MTCH2*^*F/F*^*CamKIIα-Cre*^+^ mice gain less weight on regular chow diet (Fig. 1e), despite an increase in food intake (Fig. 1f). Moreover, indirect calorimetry revealed that *MTCH2*^*F/F*^*CamKIIα-Cre*^+^ mice have increased O_2_ consumption (Fig. 1g), increased CO_2_ production (Fig. 1h), and increased heat production (Fig. 1i). In addition, *MTCH2*^*F/F*^*CamKIIα-Cre*^+^ mice display augmented nocturnal locomotor activity (resulting both from an increase in spontaneous voluntary movement and in exploratory activity; Supplementary Fig. S1h,i and j). On the other hand, the *MTCH2*^*F/F*^*CamKIIα-Cre*^+^ mice display impaired motor coordination and impaired balance in the rotarod test (Supplementary Fig. S1k), but no differences in anxiety (Supplementary Fig. S1l).

Thus, loss of forebrain MTCH2 impairs mitochondria and whole-body energy metabolism, locomotor activity, motor coordination and balance.

### *MTCH2* ^*F/F*^ *CamKIIα-Cre* ^+^ mice display hippocampal-dependent cognitive deficits

**Figure 2:**
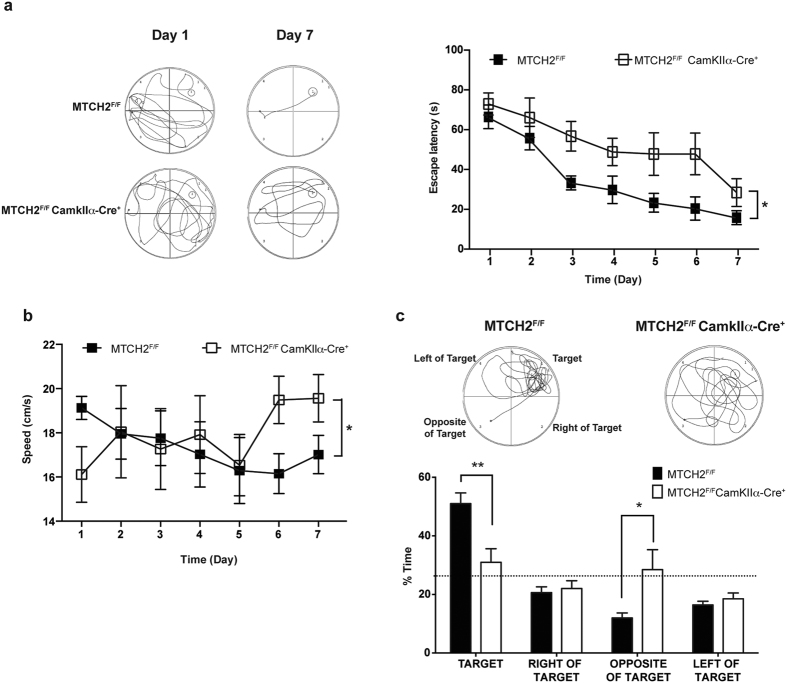
*MTCH2*^*F/F*^*CamKIIα-Cre*^+^ mice display a hippocampal-dependent cognitive deficit. (**a**) *MTCH2*^*F/F*^ *CamKIIα-Cre*^+^ mice display a learning deficit. Mice were subjected to 4 trials per day with an interval of 15 min, for 7 consecutive days (See Methods). Left panel: representative swimming paths. Right panel: Mean escape latency over time during training sessions in the water maze test. Results are presented as mean ± SEM (*p ≤ 0.05, n = 10–13 mice; two-factor with replication ANOVA was used for the statistical analysis). (**b**) *MTCH2*^*F/F*^*CamKIIα-Cre*^+^ mice show increased swimming speed. Results are presented as mean ± SEM (*p ≤ 0.05, n = 10–13 mice; two-factor with replication ANOVA). (**c**) *MTCH2*^*F/F*^*CamKIIα-Cre*^+^ mice show a spatial memory deficit. Spatial memory was evaluated using a probe test in the Morris Water Maze expressed as percentage of time spent in different zones (bottom panel) and representative swimming paths (top panel). Results are presented as mean ± SEM (*p < 0.05, **p < 0.01; n = 10–13 mice; two-tailed, unpaired, unequal variance t-test).

To assess the effect of forebrain MTCH2 loss on cognitive functions, we trained *MTCH2*^*F/F*^and *MTCH2*^*F/F*^*CamKIIα-Cre*^+^ mice to locate a hidden platform in the Morris Water Maze. As compared with *MTCH2*^*F/F*^mice, *MTCH2*^*F/F*^*CamKIIα-Cre*^+^ mice latency to locate the platform was significantly longer throughout the training process (Fig. 2a, left and right panels). Notably, the *MTCH2*^*F/F*^*CamKIIα-Cre*^+^ mice showed increased swimming speed in the last two days of training (Fig. 2b), suggesting that their improved latency to locate the platform on the last day is owing to more efficient scanning of the maze and not because the mice memorised the exact location of the platform. This is reflected also in the representative swimming paths (Fig. 2a, left panel). Memory was assessed at the end of the learning process (24 hours after the last trial) by removing the hidden platform. While the control mice spent most of their time in the quadrant where the platform had been, the *MTCH2*^*F/F*^*CamKIIα-Cre*^+^ mice spent equal time in all the quadrants of the round water maze, displaying impaired spatial memory (Fig. 2c).

### Hippocampal long term potentiation (LTP) is impaired in slices of *MTCH2* ^*F/F*^ *CamKIIα-Cre* ^+^ mice

**Figure 3:**
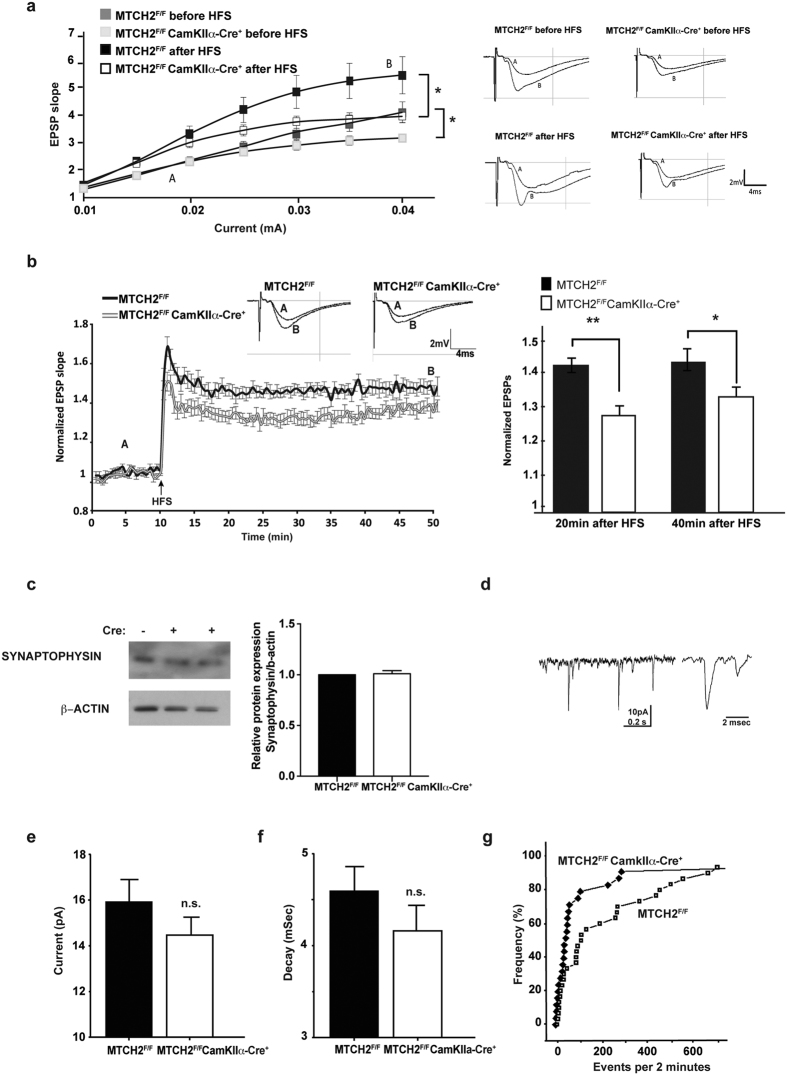
Hippocampal LTP is impaired in slices of *MTCH2*^*F/F*^*CamKIIα-Cre*^+^ mice. (**a**) Ratio of field excitatory postsynaptic potential (EPSP) slope (abscissa) to stimulus intensity (ordinate). At high-intensity (0.04 mA), input-output was significantly reduced in slices from *MTCH2*^*F/F*^*CamKIIα-Cre*^+^ mice compared to *MTCH2*^*F/F*^, both before and 40 min after LTP induction. (**b**) Normalized EPSP slopes recorded before (A) and after high-frequency stimulation (HFS, 100 Hz, 1 s, applied at arrowhead at twice of the test intensity). Representative recordings are shown above the records. Right panel: mean response change recorded in the slices at 20 and 40 minutes after the tetanic stimulation (**p < 0.01, *p < 0.05; n = 9–14 slices, from 3 mice/each genotype). (**c**) Western blot analysis of synaptophysin in primary hippocampal neurons prepared from *MTCH2*^*F/F*^(−) and *MTCH2*^*F/F*^ *CamKIIα-Cre*^+^ (+) mice. β-Actin served as a loading control. (**d**) Sample illustration of the recording (left) and an expanded image of a single mEPSC (right). (**e**) Mean sizes (in pA) of mEPSCs of *MTCH2*^*F/F*^(n = 29 cells) and *MTCH2*^*F/F*^ *CamKIIα-Cre*^+^ (n = 24 cells) from 5 independent experiments. (**f**) Decay time (in msec) of the two groups described in (**e**). (**g**) Cumulative histogram, where each cell is represented once, with the number of events per 2 minute recording time on the abscissa, and cumulative number of cells on the ordinate. There were many more *MTCH2*^*F/F*^cells with a larger number of events during the recording interval (*p* < 0.001, non-parametric test).

To directly test the ability of the hippocampus in undergoing long-term changes in responses to afferent stimulation, we conducted experiments with acute hippocampal slices using extracellular recording of population activity. At increasing stimulation intensities, the responses in CA1 region of *MTCH2*^*F/F*^*CamKIIα-Cre*^+^ mice were reduced compared to *MTCH2*^*F/F*^littermates, both before and 40 min after LTP induction (Fig. 3a, left and right panels). Tetanic stimulation caused a significantly lower potentiation in *MTCH2*^*F/F*^*CamKIIα-Cre*^+^ mice compared to *MTCH2*^*F/F*^littermates (Fig. 3b, left and right panels).

To assess whether the cognitive deficits described above are due to deficits in hippocampal neuron activity, we examined intrinsic and synaptic properties of hippocampal neurons in primary cultures. Cultured *MTCH2*^*F/F*^and *MTCH2*^*F/F*^*CamKIIα-Cre*^+^ neurons were morphologically similar (Supplementary Fig. S2a,b,c and d), established synaptic connections and retained comparable protein levels of the neuronal markers synaptophysin (Fig. 3c), glutamate transporter (vGluT1), PSD95, and glutamate receptor (GluR1) (Supplementary Fig. S2e,f and g, respectively). Recordings were made for standard 2-minute epochs from 19 *MTCH2*^*F/F*^ cells and 25 *MTCH2*^*F/F*^*CamKIIα-Cre*^+^ cells, in 5 different experiments (Fig. 3d). There were no apparent differences in input resistance and capacitance between the *MTCH2*^*F/F*^and *MTCH2*^*F/F*^*CamKIIα-Cre*^+^ cultures in response to 5mV hyperpolarizing voltage commands (data not shown). The amplitudes of miniature excitatory postsynaptic currents (mEPSCs) as well as the decay time of the spontaneous events were not different between the two groups (Fig. 3e,f). However, the *MTCH2*^*F/F*^neurons generated significantly more spontaneous events than the *MTCH2*^*F/F*^*CamKIIα-Cre*^+^ neurons, as illustrated in the cumulative histogram of rates of mEPSCs in the two groups (Fig. 3g).

Taken together, mice deficient in forebrain MTCH2 display a hippocampal-dependent cognitive deficit, which may stem from impaired hippocampal LTP and synaptic connectivity.

### *MTCH2* ^*F/F*^ *CamKIIα-Cre* ^+^ hippocampal neurons express a deficit in mitochondria motility and in mitochondria calcium handling

**Figure 4:**
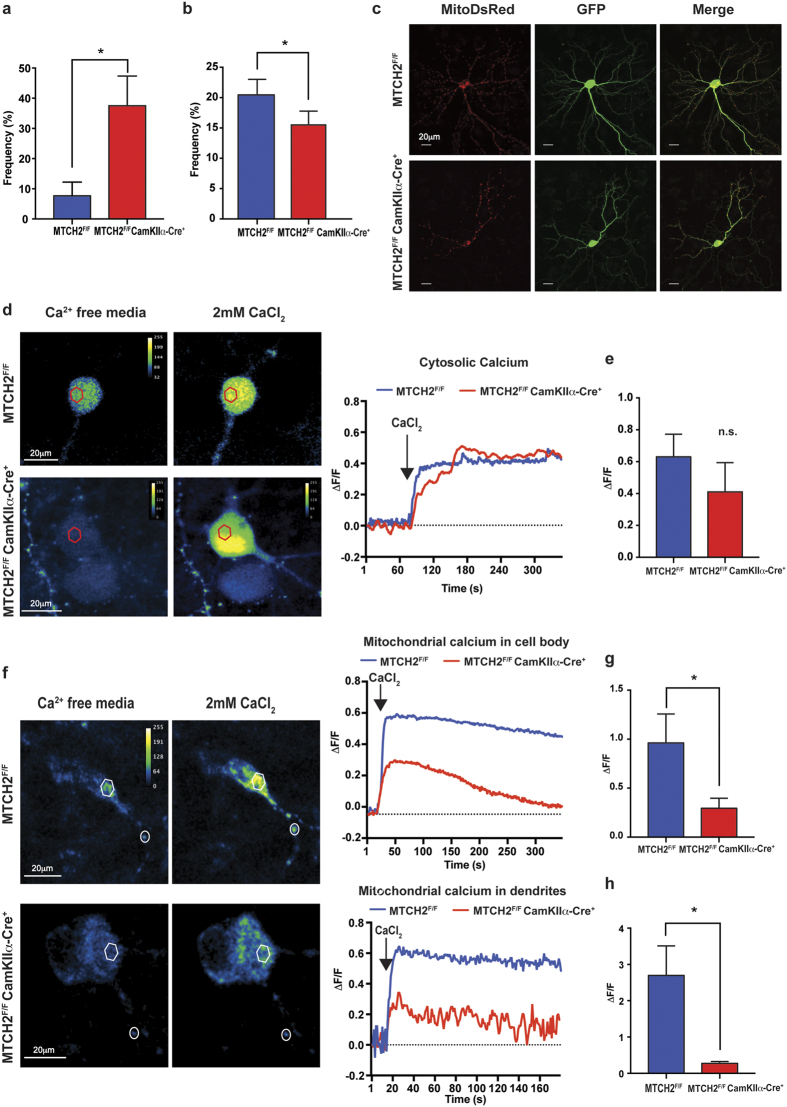
*MTCH2*^*F/F*^*CamKIIα-Cre*^+^ hippocampal neurons express a deficit in mitochondria motility and in mitochondria calcium handling. **(a)** Loss of forebrain MTCH2 decreases mitochondria motility: relative percentage of axons per cell where no movement was detected. Mean ± SEM (*p = 0.048; Unpaired t test with Welch’s correction). (**b**) Comparison of the percentage of motile mitochondria in *MTCH2*^*F/F*^ versus *MTCH2*^*F/F*^ *CamKIIα-Cre*^+^ primary hippocampal neurons. Mean ± SEM (*p = 0.039; unpaired t test with Welch’s correction). **(c)** Mitochondrial distribution in primary hippocampal cells. Neurons were co-transfected with the mitoDsRed and GFP plasmids and imaged at div10 for mitochondria distribution (left panels) and neuronal morphology (GFP; middle panels). The right panels are a merge of the left and middle panels. Scale bar, 20 μm. (**d**) No differences in cytosolic [Ca^2+^] regulation. Changes in cytosolic calcium were measured in primary hippocampal neurons by incubating the cells with Fluo-2AM. In the left panel, images of neurons displaying increase in fluorescence following addition of calcium into the bathing solution. Calcium addition to the Ca^2+^-free media (arrow, on the right panel) caused a rise in intracellular free [Ca^2+^] in both *MTCH2*^*F/F*^ and *MTCH2*^*F/F*^ *CamKIIα-Cre*^+^ cells. The red hexagons on the left panels represent the region of interested (ROI) used to generate the graphs on the right. (**e**) Averages of relative maximal fluorescence intensity of neurons loaded with Fluo-2AM. Mean ± SEM (*p* = 0.39; n = 3–5 cells/genotype; 2 independent experiments). (**f**) Reduced mitochondrial calcium uptake in *MTCH2*^*F/F*^ *CamKIIα-Cre*^+^ primary neurons. Left panel: Images of neurons transfected with mitochondria mt-RCaMP calcium sensor in Ca^2+^ free media and following the addition of calcium into the bathing solution. Right panel: upper graph represents changes in calcium in mitochondria localized in the soma of the neurons (white hexagon in the left panel); lower graph represents changes in dendritic mitochondrial calcium (white circle in the left panel). (**g**) Averages of relative maximal fluorescence intensity of mitochondria expressing mt-RCaMP in the cell body. Mean ± SEM (*p < 0.05; n = 6–10 cells/genotype; 4 independent experiments). (**h**) Averages of relative maximal fluorescence intensity of mitochondria expressing mt-RCaMP in the dendrites. Mean ± SEM (*p < 0.05; n = 3–5 mitochondria per cell; n = 6–10 cells/genotype; 4 independent experiments).

Neurons strictly depend on mitochondrial trafficking for preserving their survival and functionality^32^, and it was previously demonstrated that the size of mitochondria may have a direct impact on mitochondria motility^15^. To assess mitochondria motility, primary hippocampal neurons were transfected with MitoDsRed and confocal time lapse images were acquired. In almost 40% of the *MTCH2*^*F/F*^*CamKIIα-Cre*^+^ cells no mitochondrial motility was detected (Fig. 4a; see also movie in Supplementary material). We then analysed the behaviour of the mitochondria in axons and dendrites where movement was detected, and also found a reduction of motile mitochondria in the *MTCH2*^*F/F*^*CamKIIα-Cre*^+^ neurons as compared to the *MTCH2*^*F/F*^ ones [Fig. 4b; no differences in track duration and length were detected (Supplementary Fig. S3a,b, respectively)]. Despite this defect in motility, we did not observe a pronounced effect on mitochondria distribution (Fig. 4c). Thus, the impaired mitochondria motility is not likely to be the only cause for the altered neuronal functionality in *MTCH2*^*F/F*^*CamKIIα-Cre*^+^ hippocampal neurons.

Since calcium homeostasis plays a critical role in hippocampal-related synaptic connectivity and plasticity, and mitochondria are pivotal in buffering cellular calcium^33^, we examined the ability of mitochondria in the *MTCH2*^*F/F*^*CamKIIα-Cre*^+^ neurons to handle calcium overloads. We loaded cultured hippocampal neurons with the high affinity cytosolic calcium indicator Fluo-2AM, and the genetic mitochondrial calcium sensor RCaMP, and examined the calcium level in the cytosolic and mitochondrial compartments, in the absence of extracellular [Ca^2+^] and following its addition back to the recording medium. There was no difference in the [Ca^2+^]_c_ between *MTCH2*^*F/F*^ and *MTCH2*^*F/F*^*CamKIIα-Cre*^+^ neurons either in the baseline condition (Ca^2+^ free media) or following its addition (Fig. 4d,e).

Importantly, in the *MTCH2*^*F/F*^*CamKIIα-Cre*^+^ neurons there was a lower increase in [Ca^2+^]_m_ compared to *MTCH2*^*F/F*^ neurons following adding back calcium to the recording medium (Fig. 4f,g,h). This difference was detected both in the somatic mitochondria (Fig. 4g) and to a larger extent in the dendritic compartment (Fig. 4h). These results are consistent with the idea that mitochondria in MTCH2-deficient hippocampal neurons display a deficit in loading calcium ions into the mitochondrial matrix, and this defect might contribute to the hippocampal deficits observed in the *MTCH2*^*F/F*^*CamKIIα-Cre*^+^ mice.

## Discussion

In the present study, we demonstrate that MTCH2 is a critical player in hippocampal neuronal biology, modulating mitochondrial structure, function and motility to result in changes in cognitive functions.

Several genome-wide association studies have related the MTCH2 locus with Alzheimer’s disease (AD), suggesting that MTCH2 deregulation can contribute to the pathogenesis of the disease. One of the early symptoms of AD is loss of memory, and in this study, we indeed found that MTCH2 deficiency in the forebrain leads to hippocampal-dependent cognitive deficits already in young mice, which correlated with a reduction in synaptic connectivity and an impaired hippocampal LTP. Moreover, the decreased LTP is most likely due to the impairment we detected in the ability of mitochondria to handle calcium.

Based on previous studies we expected that loss of MTCH2, resulting in increased mitochondrial OXPHOS/metabolism would confer an advantage to the MTCH2 deficient neurons in excitability and firing^22^,^34^. However, we did not detect any apparent differences in input resistance and capacitance and actually observed a significant reduction in spontaneous synaptic activity. This reduction in mEPSCs can be attributed to a decrease in presynaptic release probability that is known to depend on energy supply and calcium homeostasis at the presynaptic terminal, which is endowed with mitochondria. Furthermore, hippocampal LTP was reduced in mice lacking MTCH2, which correlates with the impaired spatial memory when the mice were challenged in the Morris Water Maze. Notably, a similar phenotype was observed in mice deficient in brain Estrogen-related receptor γ (ERRγ), a master transcriptional regulator of many genes involved in mitochondria metabolism, including MTCH2^22^. Interestingly, in the Pei *et al*. study^22^ the decrease in learning and memory was associated with a decrease in mitochondrial OXPHOS/metabolism. Thus, balanced mitochondrial metabolism is crucial for intact memory and both reduced and increased mitochondrial metabolism may lead to similar impairments. Moreover, it should be noted that the increase in mitochondria metabolism seen in the MTCH2-deficient cells/mice can possibly be due to an attempt to compensate for defects in mitochondrial function, such as the ones we detected in motility and calcium handling.

In neurons, slowing down mitochondrial motility prevents them from properly distributing throughout the axodendritic domains to enable neuronal activity^2^. It was previously demonstrated that the size of mitochondria may have a direct impact on mitochondria motility, since hippocampal neurons expressing a defective form of the mitochondrial fission protein Drp1 (and thus mitochondria are more elongated) display accumulated mitochondria within the soma and reduced mitochondrial density in dendrites^15^. In addition, it has been shown that stimulation of neurons by excitatory doses of glutamate inhibits mitochondria motility and alters mitochondria from an elongated to a rounded shape^35^. These findings are consistent with the idea that the increase in mitochondria size/volume that occurs following loss of MTCH2, leads to the decrease in mitochondria motility. However, we did not observe a clear effect on mitochondrial distribution in the primary hippocampal neurons, and therefore the impaired motility of mitochondria might not be the only cause for the altered neuronal functionality observed in the absence of MTCH2.

In fact, we detected a defect in calcium uptake by the mitochondria in *MTCH2*^*F/F*^*CamKIIα-Cre*^+^ neurons that is likely to be related to the impaired learning and memory, lower LTP and decreased spontaneous neuron activity. Calcium homeostasis plays a critical role in hippocampal-related synaptic connectivity and plasticity, and mitochondria are pivotal in buffering cellular calcium^33^,^36^. When proper mitochondria calcium uptake is compromised, defects in cognitive functions have been reported^21^,^37^,^38^,^39^. For example, human patients carrying loss-of-function mutations in MICU1 (the regulator of the mitochondrial Ca^2+^ uniporter (MCU)), exhibit learning disability^18^. Moreover, it was recently demonstrated that deletion of the MCU in Drosophila mushroom body neurons causes memory impairment^21^. How exactly MTCH2 regulates mitochondrial calcium handling is still under investigation. MTCH2 might directly/indirectly regulate the activity of the mitochondria calcium transporters^40^,^41^ or might be part of a complex that controls calcium flow from the endoplasmic reticulum to mitochondria^42^,^43^,^44^,^45^,^46^,^47^.

Using the general forebrain *CamKIIα-Cre* model, we were able to observe additional phenotypes that seem not to be directly related to the hippocampal cognitive function. For instance, *MTCH2*^*F/F*^*CamKIIα-Cre*^+^ mice displayed increased locomotor activity that is not associated with an anxiety-related disorder. This increased locomotor activity does not correlate with better performance in behavioural tests designed for motor functions; on the contrary *MTCH2*^*F/F*^*CamKIIα-Cre*^+^ mice showed impaired coordination. Interestingly, the *MTCH2*^*F/F*^*CamKIIα-Cre*^+^ mice showed an increase in swimming speed in the last couple of days of training in the Morris Water Maze test, raising the possibility that these mice eventually located the platform by increasing their swimming speed and not by memorising its exact location. Thus, increasing locomotor activity is possibly a way for these mice to compensate for their cognitive deficits.

Notably, most of the genes controlling body mass index identified with MTCH2 in the GWA studies are highly expressed in the brain^7^,^8^,^9^,^10^,^11^,^12^. Importantly, *MTCH2*^*F/F*^*CamKIIα-Cre*^+^ mice gain 5-to-10% less weight on regular chow diet although their daily food consumption is higher. Moreover, these mice consume more oxygen and produce more CO_2_ and heat. These results indicate that the *MTCH2*^*F/F*^*CamKIIα-Cre*^+^ mice have increased whole-body metabolism, similar to our previously reported mice deficient for MTCH2 in skeletal muscle^13^. Thus, targeting MTCH2 could provide a new therapeutic avenue for metabolic disorders.

In summary, we identified forebrain MTCH2 as a pivotal regulator of hippocampal neuron metabolism and function. Our findings represent an advance in establishing the connection between mitochondrial activity and cognitive functions, with potential important contribution to our understanding of neurodegenerative diseases.

## Materials and Methods

### Mice

All methods were carried out in accordance with relevant guidelines and regulations. All experimental protocols were approved by the Weizmann Institute Animal Care and Use Committee. *MTCH2*^*F/F*^ mice were generated as described previously^6^. *CamKIIα-Cre*^+^ mice (on a pure C57BL/6 background) were kindly provided by Prof. Alon Chen (Dept. of Neurobiology, Weizmann Institute of Sciences). Mice were housed in an air-conditioned room (temperature 21 °C, relative humidity 10%) with a reversed light-dark cycle (lights on from 8 PM to 8 AM) and with ad libitum food availability and drinking water (autoclaved water). All the experiments were performed in the dark phase of the diurnal cycle and repeated with at least three independent cohorts of 10- to 16-week-old littermate female mice.

### Quantitative Real-time PCR

Total RNA was isolated from the dissected brain region using PerfectPure RNA Tissue kit (Cat #2302410, 5 Prime INC.). Samples were reverse transcribed (Cat #4368814, Applied Biosystems) and quantitative real-time PCR was performed on the cDNAs in the presence of SYBR Green (Cat #4385612, Applied Biosystems). Expression levels were determined using the comparative cycle threshold (2-ΔΔCt) method, and hypoxanthine guanine phosphoribosyl transferase (HPRT) served as a housekeeping gene. Primer sequences used for the genes tested are listed below:

MTCH2 F-5′-TGTTCACAGGCTTGACTCCA-3′

MTCH2 R-5′-CAAACTGTATAGGTGAATGGCTCT-3′

HPRT F-5′-GCAGTACAGCCCCAAAATGG-3′

HPRT R-5′-GGTCCTTTTCACCAGCAAGCT-3′

### Metabolic cages

Indirect calorimetry and food intake, as well as locomotor activity, were measured using the LabMaster/Phenomaster system (TSE Systems). The calorimetry system is an open-circuit system that determines O_2_ consumption, CO_2_ production, and heat release. Data were collected after 48 h of adaptation in acclimated singly housed mice.

### Morris Water Maze

For the acquisition phase, mice were subjected to 4 trials per day with an interval of 15 mins, for 7 consecutive days. In each trial, the mice were required to find a hidden platform located 1 cm below the water surface in a 120 cm-diameter circular pool. In the testing room, only distal visual-spatial cues for locating the hidden platform were available. The escape latency in each trial was recorded up to 90 s. Each mouse was allowed to remain on the platform for 15 s and was then removed from the maze. If the mouse did not find the platform within the 90 s, it was manually placed on the platform for 15 s. Memory was assessed 24 hours after the last trial. The escape platform was removed and mice were allowed to search for it for 1 minute; and the time spent in the different quadrants of the pool was recorded using VideoMot2 automated tracking system (Tse-System).

### Electrophysiological studies on acute hippocampal slices

Mice were rapidly decapitated with a guillotine, their brain removed and the hippocampus was sliced into transverse, 400 μm slices on a McIllwain tissue chopper, as detailed elsewhere^48^. Slices were incubated for 1.5 h in carbogenated (5% CO_2_ and 95% O_2_) ACSF at room temperature. The medium contained 124 mM NaCl, 4.4 mM KCl, 25 mM NaHCO_3_, 1 mM NaH_2_PO_4_, 2.5 mM CaCl_2_, 1.2 mM MgSO_4_ and 10 mM glucose, at pH = 7.4. Recording was made from slices that were slightly submerged in a standard chamber at 33.8–34.0 °C. Field excitatory postsynaptic potentials (EPSPs) were recorded through a glass pipette containing 0.75 M NaCl (4 MΩ). EPSPs were recorded in stratum radiatum of CA1 region. Synaptic responses were evoked by stimulation of the Schaffer collaterals. High-frequency stimulation (1 s, 100 Hz) for induction of LTP was delivered to the stimulating electrode. Before applying the tetanic stimulation, evoked EPSPs (50% of maximum amplitude) were recorded for a stable baseline period of at least 10 min. Data acquisition and off-line analysis were performed using pCLAMP 9.2 (Axon Instruments). All numerical data are expressed as mean ± SEM, and EPSP slope changes after high- and low-frequency stimulation and drug application were calculated with respect to baseline. Statistical comparisons were performed by ANOVA tests. P values of <0.05 were considered a significant difference between means.

### Primary hippocampal cultures

Primary hippocampal neurons were prepared as described^49^ with minor modification. Briefly, brains from 1–2 days old *MTCH2*^*F/F*^ and *MTCH2*^*F/F*^*CamKIIα-Cre*^+^ littermate mice were dissected in prechilled L-15 media (Cat #11415-049, Invitrogen) supplemented with 0.6% glucose (Cat #G8769, Sigma-Aldrich) and gentamycin (Cat #G1397, Sigma-Aldrich). After removal of the meninges, the dissected hippocampi were incubated in a Ca^2+^ and Mg^2+^-free HBSS (Cat #12657, Invitrogen) solution supplemented with 0.6% glucose and gentamycin (20 μg/ml) (complete HBSS) containing 0.25% trypsin (Cat #15090-046, Invitrogen), 1 mg/ml DNaseI (Cat #DN25, Sigma-Aldrich) for 30 min at 37 °C. The tissues were then washed three times with complete HBSS. The dissociated cells were re-suspended and plated in minimum essential medium (MEM) (Cat #32360-026, Invitrogen) supplemented with 0.6% glucose, 10% horse serum (NHS, Cat #16050, Invitrogen), 2% B-27 (Cat #17504-044, Invitrogen), 2 mM GlutaMaxI and gentamycin. For electrophysiological and mitochondrial motility studies, cells were plated on glass coverslip with a glial support layer.

### Culture electrophysiology

Cultures were placed on the stage of an Olympus inverted microscope and cells were imaged using phase objectives. Standard recording medium contained 129 mM NaCl, 4 mM KCl, 1 mM MgCl_2_, 2 mM CaCl_2_, 10 mM glucose, 10 mM HEPES, and pH was adjusted to 7.4 with NaOH, and osmolality to 320 mOsm with sucrose. Recording was made with patch pipettes, as previously described^50^. Voltage-clamped current signals were amplified using a Multiclamp 700B amplifier, and mEPSCs were recorded in the presence of TTX (0.5 μM) and bicuculline (10 μM). In some experiments a pressure application of high osmolality medium (addition of 300 mM sucrose to the standard recording medium) through the tip of a nearby pipette was used to facilitate release of neurotransmitters from terminals. Data were accumulated with PClamp 10 (Axon Instruments Inc) software and analyzed with Minianalysis software (Synaptosoft, Inc). Statistical comparisons were made with student’s t tests or ANOVA, using Origin software.

### Respiration measurement

For the respiration studies, the primary culture neurons were plated at 1.2 × 10^5^ cells/cm^2^ on poly-L-lysine precoated XF 24 plates (Seahorse Bioscience, Cat. #100777-004). The medium was changed the following day to Neurobasal culture medium A supplemented with B-27, 2 mM GlutaMaxI (all from Invitrogen), 0.6% glucose and gentamycin and fed every 3 days by replacing one-third of the medium with fresh media. On the third day, 4 μM of cytosine arabinoside (AraC) was added to the media to reduce the amount of glia and obtain a purer neuronal culture. Cells were cultured 6 to 7 days prior to experiments. Measurement of intact cellular respiration was performed using the Seahorse XF24 analyser (Seahorse Bioscience Inc.) and the XF Cell Mito Stress Test Kit according to the manufacturer’s instructions and as described^51^,^52^. Respiration was measured under basal conditions, and in response to the electron transport chain accelerator ionophore FCCP (Trifluorocarbonylcyanide Phenylhydrazone; 3 μM), which induces Maximal OCR (Oxygen Consumption Rate). Finally, respiration was stopped by adding the electron transport chain inhibitors Rotenone and Antimycin A (1 μM and 10 μM respectively). Values were normalised to cellular protein levels.

### Transmission Electron microscopy

Mice were anesthetised with Pental (CTS) and transcardially perfused first with ice-cold PBS and then with 3% paraformaldehyde, 2% glutaraldehyde in 0.1 M cacodylate buffer containing 5 mM CaCl_2_ (pH 7.4). After overnight fixation, brains were embedded in 7% agarose and coronal sections were prepared using a vibratome. Samples were next post-fixated in 1% osmium tetroxide supplemented with 0.5% potassium hexacyanoferrate tryhidrate and potassium dichromate in 0.1 M cacodylate (1 hr), stained with 2% uranyl acetate in water (1 hr), dehydrated in graded ethanol solutions, and embedded in Agar 100 epoxy resin (Agar Scientific). Ultrathin longitudinal sections of the CA1 region of the hippocampus (70–90 nm) were viewed and photographed with a Tecnai Spirit (FEI) transmission electron microscope operated at 120 kV and equipped with an Eagle CCD camera. Random fields of the CA1 neuronal soma were chosen, and the mitochondrial area was measured using ImageJ software.

### Volumetric analysis

One-week old primary hippocampal neurons were transfected with MitoDsRed and CamKIIα-GFP plasmids (0.5 μg each) using PolyJet™ *In Vitro* DNA Transfection Reagent (Cat #100688, SignaGen Laboratories) according to the manufacturer’s instruction for cells hard to transfect. Cells were then visualized on a 60× magnification lens (NA = 1.4) using the AX10 Carl Zeiss Microscope with a Yokogawa Spinning Disc (CSU-x1). All the images were taken under the same conditions of laser power and light exposure time. Mitochondrial 3D reconstruction was achieved using the segmentation algorithm of Imaris software (Bitplane).

### Mitochondria motility

Mitochondria motility was measured as previously described^53^ with some modification. Primary hippocampal neurons co-transfected with MitoDsRed and CamKIIα-GFP plasmids were maintained in 5% (vol/vol) CO_2_ at 37 °C and imaged for an hour by a researcher blind to the genotypes during image acquisition and data processing. Images were acquired using a 60×, 1.4-N.A. oil AX10 Carl Zeiss Microscope with a Yokogawa Spinning Disc (CSU-x1); lasers were used at 0.3–0.6% to minimize damage. Images were captured every 10 s for 3–5 min using Elements 4.0 software. Sections of axons 50–200 μm in length at least 50 μm away from the soma were selected for analysis using Imaris software (Bitplane). TimeSubstractAverage algorithm was used to remove stationary mitochondria, and each mitochondrion was manually tracked. Mitochondria were considered “mobile” if they exhibited movement faster than 0.03 μm/s and for more than 30 s. Data are presented as average of 3 independent experiments; in each experiment, at least 8 axons per sample were imaged.

### Calcium imaging

Primary hippocampal neurons were co-transfected with the mt-RCaMP plasmid coding for the mitochondrion-associated calcium sensing protein (1.2 μg^54^) and with the blue fluorescent protein (BFP) plasmid, as an indicator of cell morphology (0.5 μg), at day 6–8 after plating cells using Lipofectamine 2000 and following manufacturer’s instructions. On day 2–4 after transfection the 13 mm coverslips containing cells were removed from the incubator and in some cases, were incubated with cytosolic calcium indicator Fluo-2AM (3 μM) for 45–50 minutes at room temperature and then taken to the stage of an upright Zeiss 860 confocal microscope. The other cultures were taken to the confocal microscope without being loaded with Fluo-2AM. Cells were imaged in the standard extracellular medium, containing 2 mM CaCl_2_ and 1 mM MgCl_2_, after which being exchanged by the calcium-free medium containing 3 mM MgCl_2_, for 15 min and then switched back to the standard medium using a peristaltic pump. Imaging was conducted at the rate of 5 frames per second and analyzed using ZEN and KaleidaGraph software. Data are presented as average of 4 independent experiments.

### Western blot

Western blot analysis of mitochondrial fractions was performed using anti-MTCH2 antibodies as previously described^6^. Western blot analysis on total cell lysate for Synaptophysin was performed as described^55^. Antibodies used for loading control were anti-SDHA Abs (Cat #14715, Abcam) and Anti-β Actin Abs (Cat #8226, Abcam)

### Neuronal morphology and spine density

Primary hippocampal neurons were transfected with green fluorescent plasmid (GFP) as an indicator of cell morphology at DIV7 as described above. At DIV14 cells were fixed with 4% PFA and imaged using the Zeiss 860 confocal microscope. Neuronal morphology was evaluated using the FilamentTracer module of the Imaris software (Bitplane). Dendritic segments of 50–100 μm were selected to determine the number of spines by a researcher blind for the genotype.

### Immunofluorescence studies

For PSD95 (Cat #SC6926, Santa Cruz), vGluT1 (Cat #75-066, Antibodies Inc.) and GluR1 (Cat #SC7608, Santa Cruz) immunostaining, fixed D14 primary hippocampal neurons were permeabilized with 0.2% Triton and blocked with 20% NHS for 1 h at room temperature. The cells were then immune-labelled in PSB containing 2% NHS overnight at 4 °C. Biotin-conjugated antibodies were used to enhance the staining. Anti-Tuj1 antibody (Neuron-specific class III beta-tubulin; Cat #845502, BioLegend) was used to identify the cells. Three to five dendritic segments per cell were used to quantify the number of puncta using the plugin ITCN of ImageJ. Data are presented as an average of 3 independent experiments.

### Rotarod

The apparatus is an automatic motor-driven treadmill (SDI’s Rota Rod System) consisting of a 7.0 × 9.5 cm spindle diameter with a fall height of 44.5 cm from the center. Mice were placed on the accelerating rod programmed to reach a speed of 40 rpm in 4 minutes. The time spent on the rotating rod was recorded. Each animal was tested 5 consecutive times with a 1-minute break between trials.

### Running wheel

Mice were singly housed in standard cages equipped with a running wheel for 14 days (Colombus Instruments). Distances were recorded every 15 minutes from a counter attached to the wheel. Wheel circumference (111.76 cm) was converted to kilometers.

### Open field

The open-field test was performed in a 50 × 50 × 22 cm white box, lit by 10 lux. The mice were placed in the box for 40 min. The parameters measured in this and in the elevated plus maze tests were quantified using an automated video tracking system (VideoMot2; TSE Systems GmbH, Bad Homburg, Germany).

### Elevated plus maze

The elevated plus maze apparatus consists of a gray polyvinyl chloride maze, comprising a central part (5 × 5 cm), two opposing open arms (30.5 × 5 cm) and two opposing closed arms (30.5 × 5 × 15 cm). The apparatus was elevated at a height of 53.5 cm and the open arms were illuminated with10 lux. Mice were placed in the center, facing an open arm to initiate a 5-min session test. The time spent in the open arms, the number of entries to the open arms and the number of entries to the closed arms was measured.

### Mitochondrial DNA

Total DNA was isolated from dissected hippocampi using MasterPure DNA Purification kit (Cat #MCD85201, Epicentre). Samples were then sonicated for 5 minutes, and quantitative real-time PCR was performed as mentioned above. Primer sequences used for the genes tested are listed below:

ChrM1 F-5′-AATCAACTCGTCTATGTGGCAAAA-3′

ChrM1 R-5′-CCAGCTATCACCAAGCTCGTT-3′

COX1 F-5′-AGCCCACTTCGCCATCATAT-3′

COX1 R-5′-GCGTCGTGGTATTCCTGAAAG-3′

Cytb F-5′-ACATTGGTAATAAAGTCAGCGAAGAG-3′

Cytb R-5′-AGCGTTTTTGCTTCCCTTTTC-3′

GFAP F-5′-GGGGCAAAAGCACCAAAGAAG-3′

GFAP R-5′-GGGACAACTTGTATTGTGAGCC-3′

### Statistical analysis

Statistical analysis was performed using Graph-Pad Prism 7.0 software. In all cases, mean ± SEM is presented. The number of independent experiments is indicated in the figure or figure legend in each case. Statistical significance was determined using two-tailed Student’s t test if not else specified.

## Additional Information

**How to cite this article:** Ruggiero, A. *et al*. Loss of forebrain MTCH2 decreases mitochondria motility and calcium handling and impairs hippocampal-dependent cognitive functions. *Sci. Rep.* **7**, 44401; doi: 10.1038/srep44401 (2017).

**Publisher's note:** Springer Nature remains neutral with regard to jurisdictional claims in published maps and institutional affiliations.

## Supplementary Material

Supplementary Information

Supplementary Video 1

Supplementary Video 2

## References

[b1] Clarke D. D. & Sokoloff L. Regulation of Cerebral Metabolic Rate. Basic Neurochemistry: Molecular, Cellular and Medical Aspects(1999).

[b2] Sheng Z.-H. & Cai Q. Mitochondrial transport in neurons: impact on synaptic homeostasis and neurodegeneration. Nat. Rev. Neurosci. 13, 77–93 (2012). 22218207 10.1038/nrn3156 PMC4962561

[b3] Mattson M. P., Gleichmann M. & Cheng A. Mitochondria in neuroplasticity and neurological disorders. Neuron 60, 748–66 (2008). 19081372 10.1016/j.neuron.2008.10.010 PMC2692277

[b4] Obashi K. & Okabe S. Regulation of mitochondrial dynamics and distribution by synapse position and neuronal activity in the axon. Eur. J. Neurosci. 38, 2350–2363 (2013). 23725294 10.1111/ejn.12263

[b5] Safiulina D. . Energetic and Dynamic: How Mitochondria Meet Neuronal Energy Demands. PLoS Biol. 11, e1001755 (2013). 24391475 10.1371/journal.pbio.1001755 PMC3876985

[b6] Zaltsman Y. . MTCH2/MIMP is a major facilitator of tBID recruitment to mitochondria. Nat. Cell Biol. 12, 553–62 (2010). 20436477 10.1038/ncb2057 PMC4070879

[b7] Willer C. J. . Six new loci associated with body mass index highlight a neuronal influence on body weight regulation. Nat. Genet. 41, 25–34 (2009). 19079261 10.1038/ng.287 PMC2695662

[b8] Heid I. M. . Meta-analysis identifies 13 new loci associated with waist-hip ratio and reveals sexual dimorphism in the genetic basis of fat distribution. Nat. Genet. 42, 949–60 (2010). 20935629 10.1038/ng.685 PMC3000924

[b9] Mei H. . Longitudinal replication studies of GWAS risk SNPs influencing body mass index over the course of childhood and adulthood. PLoS One 7, e31470 (2012). 22355368 10.1371/journal.pone.0031470 PMC3280302

[b10] Ng M. C. Y. . Implication of genetic variants near NEGR1, SEC16B, TMEM18, ETV5/DGKG, GNPDA2, LIN7C/BDNF, MTCH2, BCDIN3D/FAIM2, SH2B1, FTO, MC4R, and KCTD15 with obesity and type 2 diabetes in 7705 Chinese. J. Clin. Endocrinol. Metab. 95, 2418–25 (2010). 20215397 10.1210/jc.2009-2077

[b11] Bauer F. . Obesity genes identified in genome-wide association studies are associated with adiposity measures and potentially with nutrient-specific food preference. Am. J. Clin. Nutr. 90, 951–9 (2009). 19692490 10.3945/ajcn.2009.27781

[b12] Cornelis M. C. . Obesity susceptibility loci and uncontrolled eating, emotional eating and cognitive restraint behaviors in men and women. Obesity (Silver Spring) 22, E135–41 (2014). 23929626 10.1002/oby.20592 PMC3858422

[b13] Buzaglo-Azriel L. . Loss of Muscle MTCH2 Increases Whole-Body Energy Utilization and Protects from Diet-Induced Obesity. Cell Rep. 14, 1602–10 (2016). 26876167 10.1016/j.celrep.2016.01.046

[b14] Maryanovich M. . An MTCH2 pathway repressing mitochondria metabolism regulates haematopoietic stem cell fate. Nat. Commun. 6, 7901 (2015). 26219591 10.1038/ncomms8901

[b15] Li Z., Okamoto K.-I., Hayashi Y. & Sheng M. The Importance of Dendritic Mitochondria in the Morphogenesis and Plasticity of Spines and Synapses. Cell 119, 873–887 (2004). 15607982 10.1016/j.cell.2004.11.003

[b16] Santos R. X. . Alzheimer’s disease: diverse aspects of mitochondrial malfunctioning. Int. J. Clin. Exp. Pathol. 3, 570–81 (2010). 20661404 PMC2907118

[b17] Tang F.-L. . VPS35 Deficiency or Mutation Causes Dopaminergic Neuronal Loss by Impairing Mitochondrial Fusion and Function. Cell Rep. 12, 1631–43 (2015). 26321632 10.1016/j.celrep.2015.08.001 PMC4565770

[b18] Logan C. V. . Loss-of-function mutations in MICU1 cause a brain and muscle disorder linked to primary alterations in mitochondrial calcium signaling. Nat. Genet. 46, 188–193 (2013). 24336167 10.1038/ng.2851

[b19] Levy M., Faas G. C., Saggau P., Craigen W. J. & Sweatt J. D. Mitochondrial Regulation of Synaptic Plasticity in the Hippocampus. J. Biol. Chem. 278, 17727–17734 (2003). 12604600 10.1074/jbc.M212878200

[b20] Finsterer J. Cognitive dysfunction in mitochondrial disorders. Acta Neurol. Scand. 126, 1–11 (2012). 22335339 10.1111/j.1600-0404.2012.01649.x

[b21] Drago I. & Davis R. L. Inhibiting the Mitochondrial Calcium Uniporter during Development Impairs Memory in Adult Drosophila. CellReports 16, 2763–2776 (2016). 10.1016/j.celrep.2016.08.017 PMC5045571 27568554

[b22] Pei L. . Dependence of hippocampal function on ERRγ-regulated mitochondrial metabolism. Cell Metab. 21, 628–36 (2015). 25863252 10.1016/j.cmet.2015.03.004 PMC4393848

[b23] Escott-Price V. . Gene-wide analysis detects two new susceptibility genes for Alzheimer’s disease. PLoS One 9, e94661 (2014). 24922517 10.1371/journal.pone.0094661 PMC4055488

[b24] Karch C. M., Ezerskiy L. A., Bertelsen S. & Goate A. M. Alzheimer’s Disease Risk Polymorphisms Regulate Gene Expression in the ZCWPW1 and the CELF1 Loci. PLoS One 11, e0148717 (2016). 26919393 10.1371/journal.pone.0148717 PMC4769299

[b25] Allen M. . Late-onset Alzheimer disease risk variants mark brain regulatory loci. Neurol. Genet. 1, e15 (2015). 27066552 10.1212/NXG.0000000000000012 PMC4807909

[b26] Yao J. . Mitochondrial bioenergetic deficit precedes Alzheimer’s pathology in female mouse model of Alzheimer’s disease. Proc. Natl. Acad. Sci. USA 106, 14670–5 (2009). 19667196 10.1073/pnas.0903563106 PMC2732886

[b27] García-Escudero V. . Deconstructing mitochondrial dysfunction in Alzheimer disease. Oxid. Med. Cell. Longev. 2013, 162152 (2013). 23840916 10.1155/2013/162152 PMC3693159

[b28] Moreira P. I., Carvalho C., Zhu X., Smith M. A. & Perry G. Mitochondrial dysfunction is a trigger of Alzheimer’s disease pathophysiology. Biochim. Biophys. Acta - Mol. Basis Dis. 1802, 2–10 (2010). 10.1016/j.bbadis.2009.10.006 19853658

[b29] Lein E. S. . Genome-wide atlas of gene expression in the adult mouse brain. Nature 445, 168–176 (2006). 17151600 10.1038/nature05453

[b30] Gene Detail:: Allen Brain Atlas: Mouse Brain. Available at: http://mouse.brain-map.org/gene/show/35708 (Accessed: 2nd June 2016).

[b31] Dragatsis I. & Zeitlin S. CaMKIIalpha-Cre transgene expression and recombination patterns in the mouse brain. Genesis 26, 133–5 (2000). 10686608 10.1002/(sici)1526-968x(200002)26:2<133::aid-gene10>3.0.co;2-v

[b32] Schwarz T. L. Mitochondrial trafficking in neurons. Cold Spring Harb. Perspect. Biol. 5, a011304 (2013). 23732472 10.1101/cshperspect.a011304 PMC3660831

[b33] Rizzuto R., De Stefani D., Raffaello A. & Mammucari C. Mitochondria as sensors and regulators of calcium signalling. Nat. Rev. Mol. Cell Biol. 13, 566–578 (2012). 22850819 10.1038/nrm3412

[b34] Marosi K. & Mattson M. P. BDNF mediates adaptive brain and body responses to energetic challenges. Trends Endocrinol. Metab. 25, 89–98 (2014). 24361004 10.1016/j.tem.2013.10.006 PMC3915771

[b35] Rintoul G. L., Filiano A. J., Brocard J. B., Kress G. J. & Reynolds I. J. Glutamate decreases mitochondrial size and movement in primary forebrain neurons. J. Neurosci. 23, 7881–8 (2003). 12944518 10.1523/JNEUROSCI.23-21-07881.2003 PMC6740596

[b36] Brown M. R., Sullivan P. G. & Geddes J. W. Synaptic mitochondria are more susceptible to Ca2+ overload than nonsynaptic mitochondria. J. Biol. Chem. 281, 11658–68 (2006). 16517608 10.1074/jbc.M510303200

[b37] Hart L., Rauch A., Carr A. M., Vermeesch J. R. & O’Driscoll M. LETM1 haploinsufficiency causes mitochondrial defects in cells from humans with Wolf-Hirschhorn syndrome: implications for dissecting the underlying pathomechanisms in this condition. Dis. Model. Mech. 7, 535–45 (2014). 24626991 10.1242/dmm.014464 PMC4007405

[b38] Jeon D. . Enhanced learning and memory in mice lacking Na+/Ca2+ exchanger 2. Neuron 38, 965–76 (2003). 12818181 10.1016/s0896-6273(03)00334-9

[b39] Weeber E. J. . The role of mitochondrial porins and the permeability transition pore in learning and synaptic plasticity. J. Biol. Chem. 277, 18891–7 (2002). 11907043 10.1074/jbc.M201649200

[b40] Gunter T. E., Buntinas L., Sparagna G., Eliseev R. & Gunter K. Mitochondrial calcium transport: mechanisms and functions. Cell Calcium 28, 285–296 (2000). 11115368 10.1054/ceca.2000.0168

[b41] del Arco A., Contreras L., Pardo B. & Satrustegui J. Calcium regulation of mitochondrial carriers. Biochim. Biophys. Acta - Mol. Cell Res. 1863, 2413–2421 (2016). 10.1016/j.bbamcr.2016.03.024 27033520

[b42] Zampese E. . Presenilin 2 modulates endoplasmic reticulum (ER)-mitochondria interactions and Ca2+ cross-talk. Proc. Natl. Acad. Sci. USA 108, 2777–82 (2011). 21285369 10.1073/pnas.1100735108 PMC3041131

[b43] Simmen T. . PACS-2 controls endoplasmic reticulum–mitochondria communication and Bid-mediated apoptosis. EMBO J. 24, 717–729 (2005). 15692567 10.1038/sj.emboj.7600559 PMC549619

[b44] de Brito O. M. & Scorrano L. Mitofusin 2 tethers endoplasmic reticulum to mitochondria. Nature 456, 605–610 (2008). 19052620 10.1038/nature07534

[b45] Szabadkai G. . Chaperone-mediated coupling of endoplasmic reticulum and mitochondrial Ca2+ channels. J. Cell Biol. 175, 901–11 (2006). 17178908 10.1083/jcb.200608073 PMC2064700

[b46] Iwasawa R., Mahul-Mellier A.-L., Datler C., Pazarentzos E. & Grimm S. Fis1 and Bap31 bridge the mitochondria-ER interface to establish a platform for apoptosis induction. EMBO J 30, 556–68 (2011). 21183955 10.1038/emboj.2010.346 PMC3034017

[b47] Cerqua C. . Trichoplein/mitostatin regulates endoplasmic reticulum-mitochondria juxtaposition. EMBO Rep. 11, 854–60 (2010). 20930847 10.1038/embor.2010.151 PMC2966954

[b48] Maggio N. & Segal M. Striking Variations in Corticosteroid Modulation of Long-Term Potentiation along the Septotemporal Axis of the Hippocampus. J. Neurosci. 27, 5757–5765 (2007). 17522319 10.1523/JNEUROSCI.0155-07.2007 PMC6672761

[b49] Segal M. & Korkotian E. Endoplasmic reticulum calcium stores in dendritic spines. Front. Neuroanat. 8, 64 (2014). 25071469 10.3389/fnana.2014.00064 PMC4089118

[b50] Vlachos A. . Synaptopodin Regulates Plasticity of Dendritic Spines in Hippocampal Neurons. J. Neurosci. 29, 1017–1033 (2009). 19176811 10.1523/JNEUROSCI.5528-08.2009 PMC6665122

[b51] Ribeiro S. M., Giménez-Cassina A. & Danial N. N. Measurement of mitochondrial oxygen consumption rates in mouse primary neurons and astrocytes. Methods Mol. Biol. 1241, 59–69 (2015). 25308488 10.1007/978-1-4939-1875-1_6

[b52] Giménez-Cassina A. . BAD-dependent regulation of fuel metabolism and K(ATP) channel activity confers resistance to epileptic seizures. Neuron 74, 719–30 (2012). 22632729 10.1016/j.neuron.2012.03.032 PMC3361694

[b53] Misgeld T., Kerschensteiner M., Bareyre F. M., Burgess R. W. & Lichtman J. W. Imaging axonal transport of mitochondria *in vivo*. Nat. Methods 4, 559–561 (2007). 17558414 10.1038/nmeth1055

[b54] Akerboom J. . Genetically encoded calcium indicators for multi-color neural activity imaging and combination with optogenetics. Front. Mol. Neurosci. 6, 2 (2013). 23459413 10.3389/fnmol.2013.00002 PMC3586699

[b55] Kim Y.-G. & Lee Y.-I. Differential Expressions of Synaptogenic Markers between Primary Cultured Cortical and Hippocampal Neurons. Exp. Neurobiol. 21, 61 (2012). 22792026 10.5607/en.2012.21.2.61 PMC3381213

