# Supplementary material for: Loss of forebrain MTCH2 decreases mitochondria motility and calcium handling and impairs hippocampal-dependent cognitive functions
Source: Sci Rep. 2017 Mar 9;7:44401. doi: 10.1038/srep44401 (PMC5343590; doi:10.1038/srep44401)

Loss of forebrain MTCH2 decreases mitochondrial motility and calcium handling and impairs hippocampal-dependent cognitive functions

Antonella Ruggiero[1], Etay Aloni[2], Eduard Korkotian[2], Yehudit Zalstman[1], Efrat Oni-Biton[2], Yael Kuperman[3], Michael Tsoory[3], Liat Shachnai[1], Smadar Levin-Zaidman[4], Ori Brenner[3], Menahem Segal[2*], and Atan Gross[1*]

Departments of Biological Regulation[1], Neurobiology[2], Veterinary Resources[3], and Chemical Research Support[4], Weizmann Institute of Science, Rehovot 76100, Israel

[*] To whom correspondence should be addressed:

Atan Gross, Ph.D.

Tel: +972-8-9343656

Fax: +972-8-9344116

Email: atan.gross@weizmann.ac.il

Menahem Segal, Ph.D.

Tel: +972-8-9342553

Fax: +972-8-9344140

Email: menahem.segal@weizmann.ac.il

Supplemental figure legends

Figure S1. *MTCH2$^{F/F}$ CamKIIα-Cre^{+}* mice display increased mitochondria respiration and size/volume, increased locomotor activity, but impaired motor coordination and balance

(**a**) *MTCH2$^{F/F}$ CamKIIα-Cre^{+}* mice have degenerative changes in the seminiferous tubules due to decreased MTCH2 expression. Histological analysis of the testis. Top panels (A,B; *MTCH2$^{F/F}$*): Normal testicular parenchyma. Many seminiferous tubules contain relatively mature stages of spermatozoa. Bottom panels (C,D; *MTCH2$^{F/F}$ CamKIIα-Cre^{+}*): Degenerative changes in seminiferous tubules. Macrophages are present in the lumen (arrowheads) and there are scattered necrotic/apoptotic cells. The number of mature spermatozoa is dramatically reduced. A,C: x40; B,D: x20. H&E staining (A-D**).**

(**b**) RT-PCR analysis shows that MTCH2 mRNA levels are decreased in the testis of *MTCH2$^{F/F}$ CamKIIα-Cre^{+}* mice.

(**c**) Representative seahorse experiment: normalised oxygen consumption rate (OCR) measured in real-time indicating basal and maximal respiration rates in XF media containing 10mM Pyruvate (as indicated by OCR before and after injection of FCCP).

(**d**) Representative EM pictures of CA1 hippocampal neurons. The area occupied by the nucleus was subtracted from the total area of the cell in order to estimate cytosolic area. Mitochondria (black arrows) were manually counted and the area was evaluated using ImageJ. Scale bars: 2μm in the main images and 500nm in the insets.

(**e**) Mitochondrial area frequency distribution. *MTCH2$^{F/F}$ CamKIIα-Cre^{+}* hippocampi have mitochondria with considerably larger area compared to *MTCH2$^{F/F}$* hippocampi due to the presence of a larger population of larger mitochondria. The data represent

mean ± SEM (*$p \leq 0.05$, ***$p \leq 0.001$; n=184-386 mitochondria/each mouse, 2-way ANOVA Fisher LSD test for multiple comparison).

(**f**) Mitochondrial density indicates the number of mitochondria per μm^2 of cytosol. The data represent mean ± SEM.

(**g**) mtDNA was extracted from 5 mice/each genotype and ChrM, COX1 and Cytb (mitochondrial-encoded genes) expression was measured by RT-PCR relative to HPRT. GFAP served as a control of a nuclear encoded gene. The data represent mean ± SEM.

(**h**) *MTCH2*$^{F/F}$ *CamKII*α*-Cre*$^{+}$ mice show increased locomotor activity (Insert: Daily locomotor activity). The data represent mean ± SEM (*$p \leq 0.05$, n=8 mice). The light and dark cycles are denoted by horizontal white and black bars, respectively.

(**i**) *MTCH2*$^{F/F}$ *CamKII*α*-Cre*$^{+}$ mice display an increase in spontaneous voluntary movement using running wheels (daily running distance). The data represent mean ± SEM (*$p \leq 0.05$; n=19-22 mice).

(**j**) *MTCH2*$^{F/F}$ *CamKII*α*-Cre*$^{+}$ mice display an increase in exploratory activity. The data represent mean ± SEM (*$p \leq 0.05$; n=7; 2-way ANOVA repeated measurement).

(**k**) *MTCH2*$^{F/F}$ *CamKII*α*-Cre*$^{+}$ mice show impaired motor coordination and balance on the rotarod. The data represent mean ± SEM (****$p \leq 0.001$, n=23 mice).

(**l**) *MTCH2*$^{F/F}$ *CamKII*α*-Cre*$^{+}$ mice do not display an increase in anxiety. Left panel: Elevated plus maze: Total distance was significantly elevated. The total amount of visits in the open and the close arm was higher. Therefore, to evaluate anxiety, the number of visits in the open arm (middle panel), as well as the distance covered (right

panel) were normalized to the total visits and distance showing no differences. The data represent mean ± SEM (**$p \leq 0.01$, n=19-23 mice).

Figure S2. No differences in neuronal morphology, spine density and synaptic markers

(**a**) Neurons were transfected with a GFP plasmid and imaged at DIV14. Neuronal morphology was evaluated using FilamentTracer module of Imaris software (Representative pictures are presented in the left and middle panels; Scale bar, 40μm). Right panel: No significant differences in the total number of branches were detected between the *MTCH2*$^{F/F}$ and *MTCH2*$^{F/F}$ *CamKII*α*-Cre*$^{+}$ primary hippocampal neurons. The data represent mean ± SEM (n=10 cells/genotype).

(**b**) Dendritic length frequency distribution. No significant differences were detected between the *MTCH2*$^{F/F}$ and *MTCH2*$^{F/F}$ *CamKII*α*-Cre*$^{+}$ primary hippocampal neurons. The data represent mean ± SEM (n=10 cell/genotype; 2 way-ANOVA Repeated measurements).

(**c**) Sholl analysis plot showing numbers of dendritic crossings along the Sholl rings as a function of distance from soma. No significant differences were detected between the *MTCH2*$^{F/F}$ and *MTCH2*$^{F/F}$ *CamKII*α*-Cre*$^{+}$ primary hippocampal neurons. The data represent mean ± SEM (n=10 cells/genotype; 2 way-ANOVA, Repeated Measurements;).

(**d**) Dendritic segments of 50-100 μm in length were used to count the number of spines from primary hippocampal neurons transfected with GFP (Representative images of dendritic segments are presented in the left panels; Scale bar, 10μm). Right

panel: No significant differences were detected between the *MTCH2*$^{F/F}$ and *MTCH2*$^{F/F}$ *CamKII*α*-Cre*$^{+}$ neurons. The data represent mean ± SEM (n=21-24 segments from 10 neurons/genotype).

(**e**) Primary hippocampal neurons were co-stained with antibodies to the neuronal marker vGluT1 and to Tuj1 (representative pictures are presented in the top panels). Bottom panel: The number of vGluT1 puncta per dendritic area was evaluated using the ITCN plugin of ImageJ. No significant differences were detected between the *MTCH2*$^{F/F}$ and *MTCH2*$^{F/F}$ *CamKII*α*-Cre*$^{+}$ neurons. The data represent the mean ± SEM (n=33-39 dendritic segments)

(**f**) Primary hippocampal neurons were co-stained with antibodies to the neuronal marker PSD95 and to Tuj1 (representative pictures are presented in the top panels). Bottom panel: The number of puncta per dendritic area was evaluated as in (e). No significant differences were detected between the *MTCH2*$^{F/F}$ and *MTCH2*$^{F/F}$ *CamKII*α*-Cre*$^{+}$ neurons. The data represent the mean ± SEM (n=60 dendritic segments)

(**g**) Primary hippocampal neurons were co-stained with antibodies to the neuronal marker GluR1 and to Tuj1 (representative pictures are presented in the top panels). Bottom panel: The number of puncta per dendritic area was evaluated as in (e). No significant differences were detected between the *MTCH2*$^{F/F}$ and *MTCH2*$^{F/F}$ *CamKII*α*-Cre*$^{+}$ neurons. The data represent the mean ± SEM (n=45 dendritic segments)

Figure S3. No differences in track length and duration of mitochondrial movement

(**a**) Mitochondrial track duration. The data represent mean ± SEM.

(**b**) Mitochondrial track length. The data represent mean ± SEM.

Supplementary movies

(a) Representative time-lapse images of mitochondria moving in *MTCH2*$^{F/F}$ primary hippocampal neurons. Primary hippocampal neurons were co-transfected with MitoDsRed and CamKIIα-GFP plasmids. Images were captured every 10s for 3-5 min while cells were maintained in 5% (vol/vol) CO_2 at 37°C.

(b) Representative time-lapse images of mitochondria moving in *MTCH2*$^{F/F}$ *CamKIIα-Cre*$^{+}$ primary hippocampal neurons. In the dendrites of *MTCH2*$^{F/F}$ *CamKIIα-Cre*$^{+}$ primary hippocampal neurons the amount of stationary mitochondria is significantly higher compare to the *MTCH2*$^{F/F}$.

Gross_Fig. S1

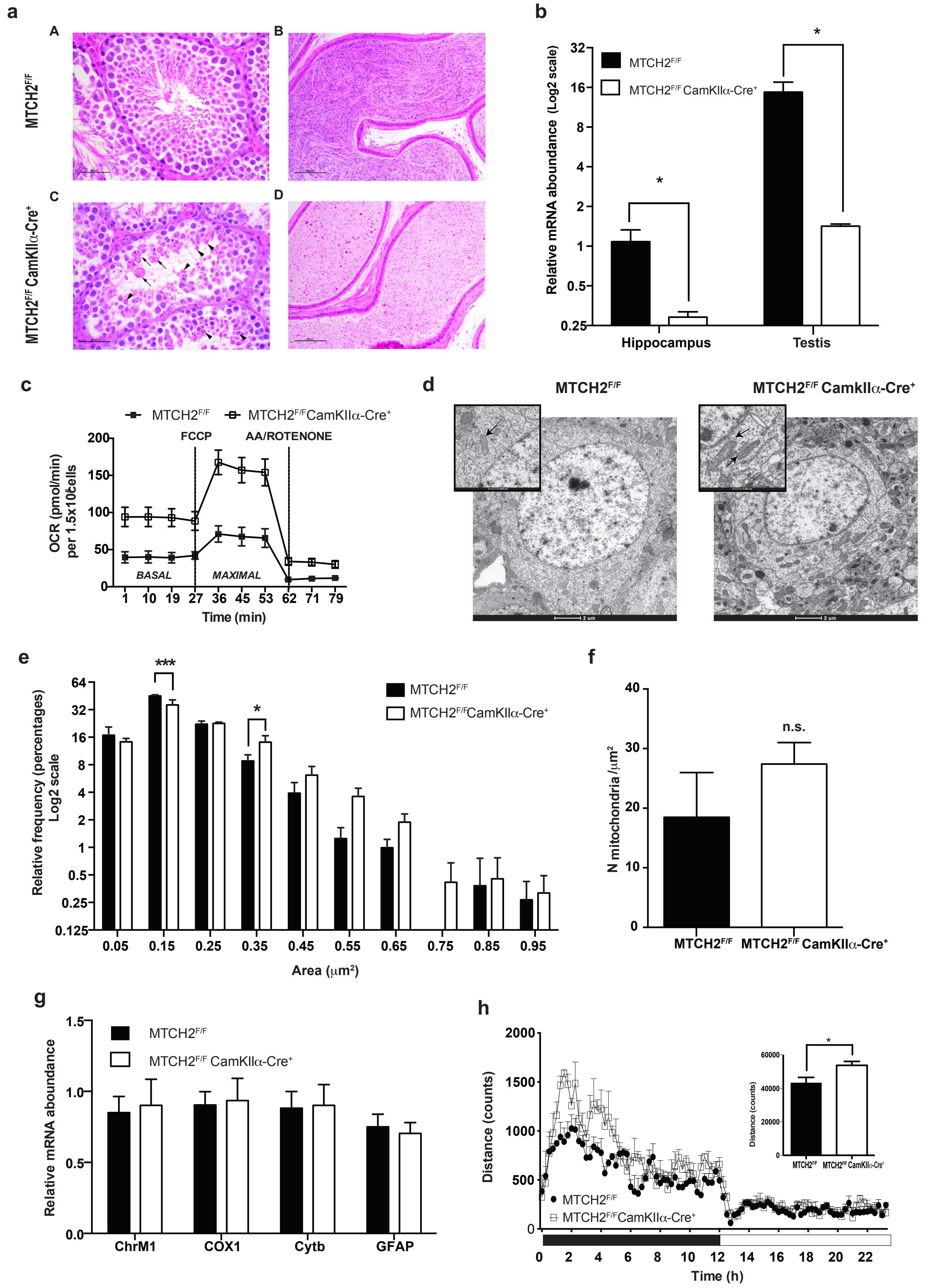
a
A
B
C
D
MTCH2F/F
MTCH2F/F CamKIIα-Cre+
b
Relative mRNA aboundance (Log2 scale)
MTCH2F/F
MTCH2F/F CamKIIα-Cre+
Hippocampus
Testis
c
MTCH2F/F
MTCH2F/FCamKIIα-Cre+
FCCP
AA/ROTENONE
OCR (pmol/min) per 1.5x10⁴cells
BASAL
MAXIMAL
Time (min)
d
MTCH2F/F
MTCH2F/F CamkIIα-Cre+
2 um
e
Relative frequency (percentages) Log2 scale
MTCH2F/F
MTCH2F/FCamKIIα-Cre+
Area (μm²)
f
N mitochondria /μm²
n.s.
MTCH2F/F
MTCH2F/F CamKIIα-Cre+
g
Relative mRNA aboundance
MTCH2F/F
MTCH2F/F CamKIIα-Cre+
ChrM1
COX1
Cytb
GFAP
h
Distance (counts)
MTCH2F/F
MTCH2F/FCamKIIα-Cre+
Time (h)
Distance (counts)

Gross_Fig. S1 continued

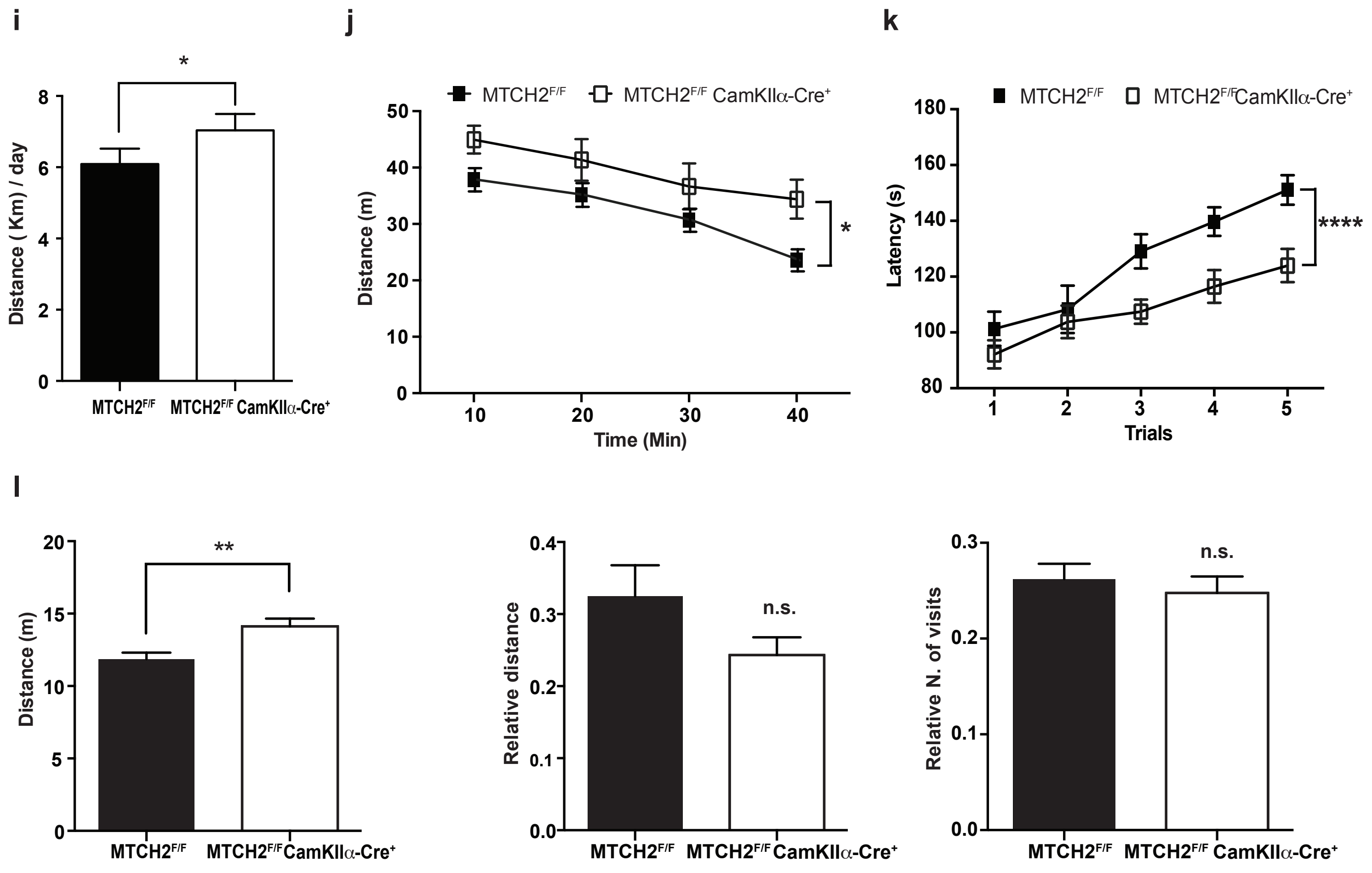

Gross_Fig. S2

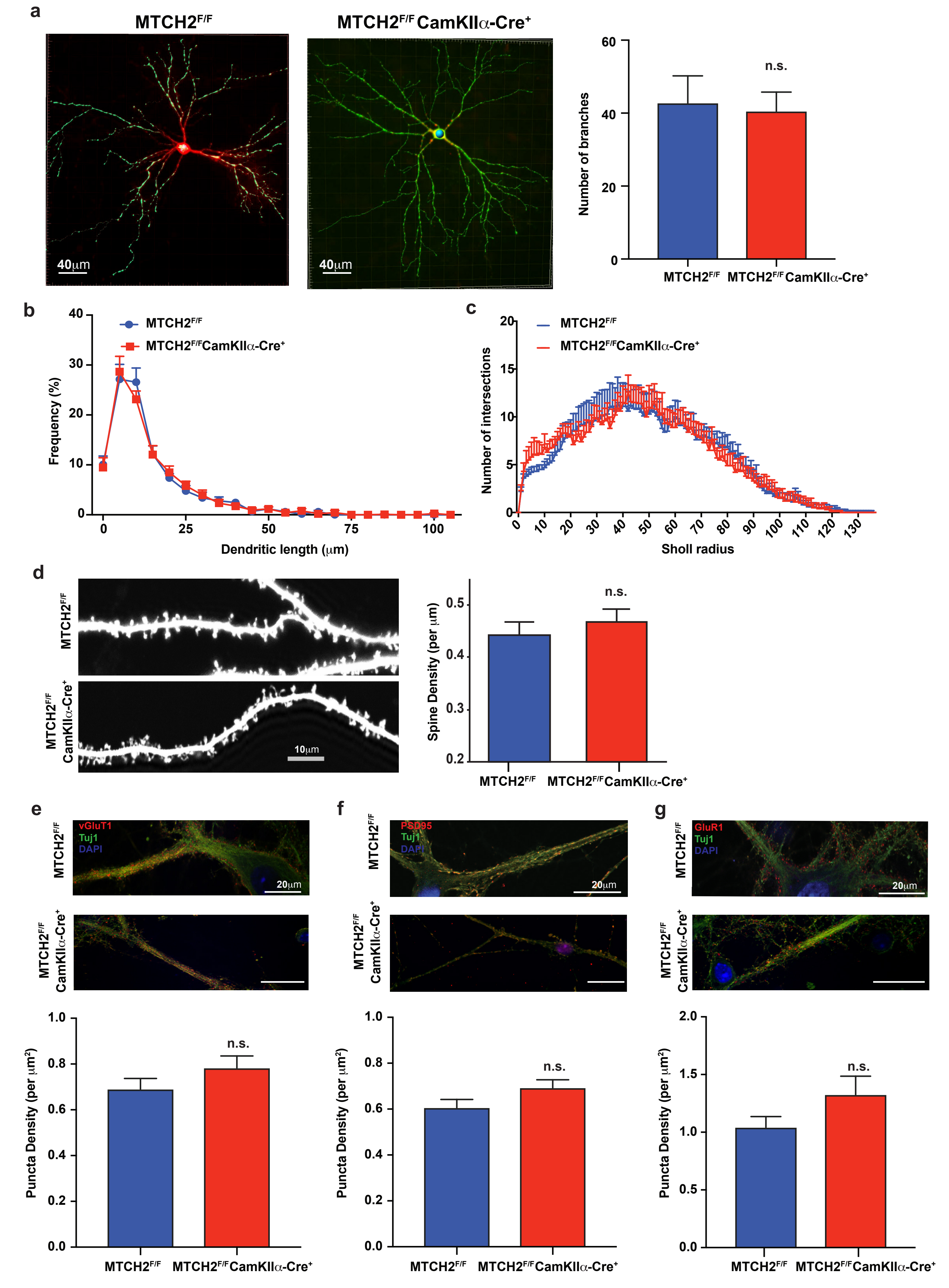
a
MTCH2F/F
MTCH2F/F CamKIIα-Cre+
40μm
40μm
Number of branches
60
40
20
0
n.s.
MTCH2F/F
MTCH2F/F CamKIIα-Cre+
b
MTCH2F/F
MTCH2F/FCamKIIα-Cre+
Frequency (%)
Dendritic length (μm)
c
Number of intersections
Sholl radius
d
MTCH2F/F
MTCH2F/F CamKIIα-Cre+
10μm
Spine Density (per μm)
e
vGluT1
Tuj1
DAPI
20μm
f
PSD95
g
GluR1
Puncta Density (per μm²)

Gross_Fig. S3

a

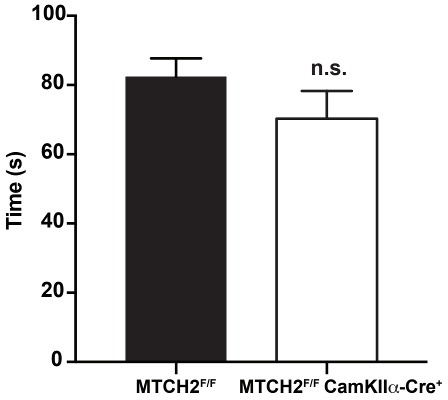

b

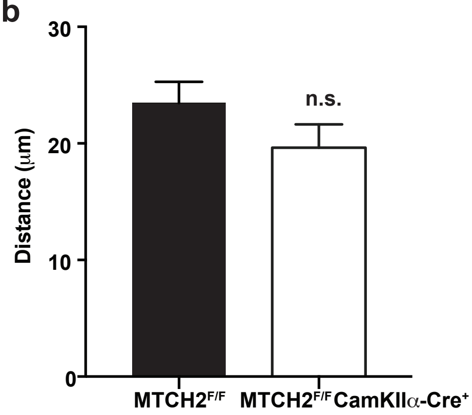

Supplement: Supplementary Information [file srep44401-s1.pdf]
